# Supplementary material for: Interspecies nutrient extraction and toxin delivery between bacteria
Source: Nat Commun. 2017 Aug 22;8:315. doi: 10.1038/s41467-017-00344-7 (PMC5566331; doi:10.1038/s41467-017-00344-7)
Supplement: Supplementary file 1 — Supplementary Information [file 41467_2017_344_MOESM1_ESM.pdf]

**Description of Supplementary Files**

File name: Supplementary Information
Description: Supplementary figures, supplementary tables and supplementary references.

File name: Peer review file

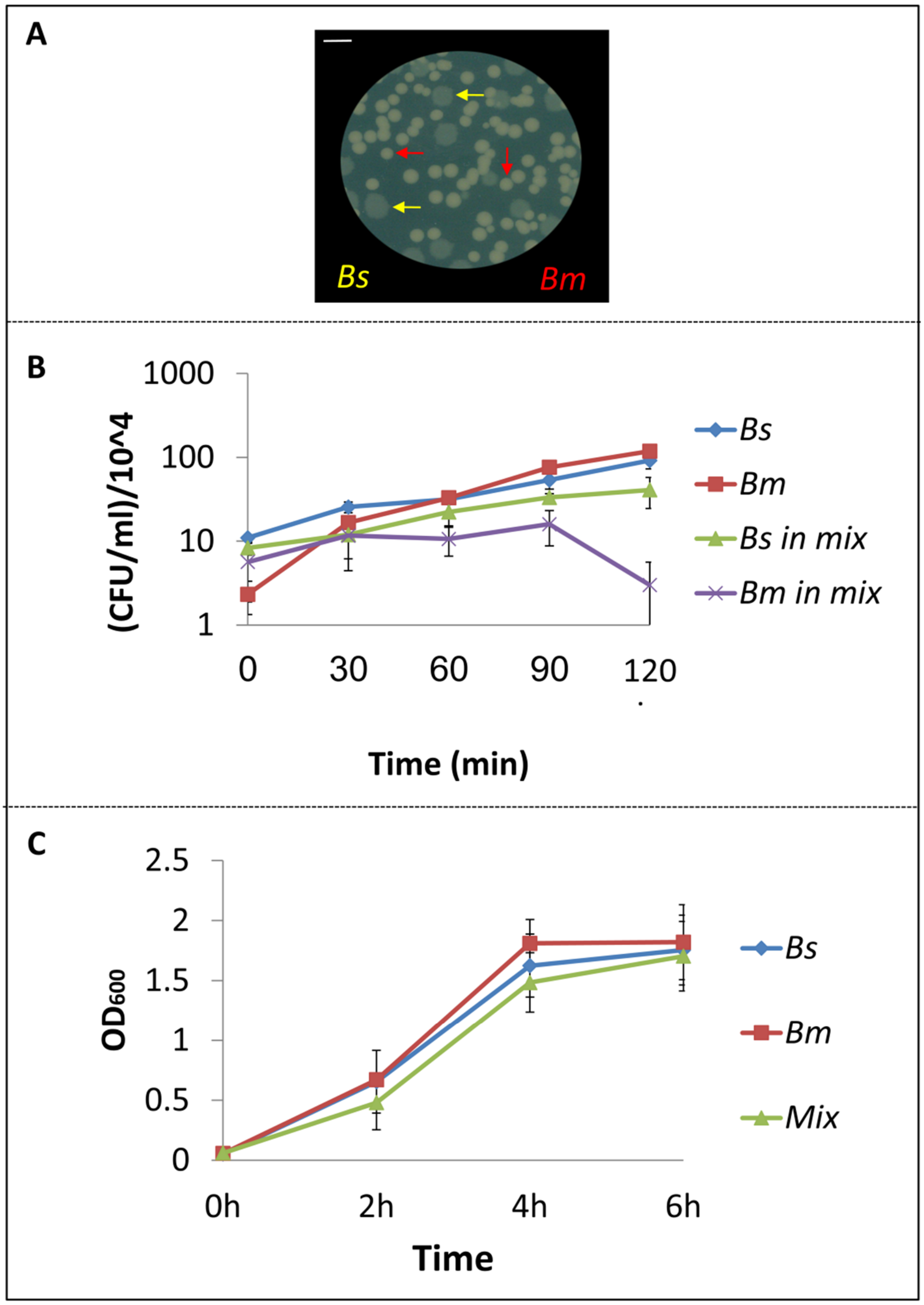


**Supplementary Figure 1: Growth characteristics of *Bs* and *Bm***

(A) *Bs* (PY79) and *Bm* (OS2) were grown separately to $OD_{600}$ 0.5, diluted to $10^{-3}$, then mixed and immediately plated on LB plate. Cells were incubated ON at 37°C. Shown is

an image of a plate containing a mixture of *Bs* (yellow arrows) and *Bm* (red arrows) colonies. Scale bar represents 1 cm.

(B) *Bs* (PY79) and *Bm* (OS2) were grown separately or in a mixture in LB medium at 37°C. Cells were plated for CFUs at the indicated time points. *Bs* and *Bm* were differentiated by colony morphology as seen in (A). Each point represents an average value and SD of three independent experiments.

(C) *Bs* (PY79) and *Bm* (OS2) were grown separately or in a mixture in LB medium at 37°C. Cell density ($OD_{600}$) was examined at the indicated time points. Each point represents an average value and SD of three independent experiments.

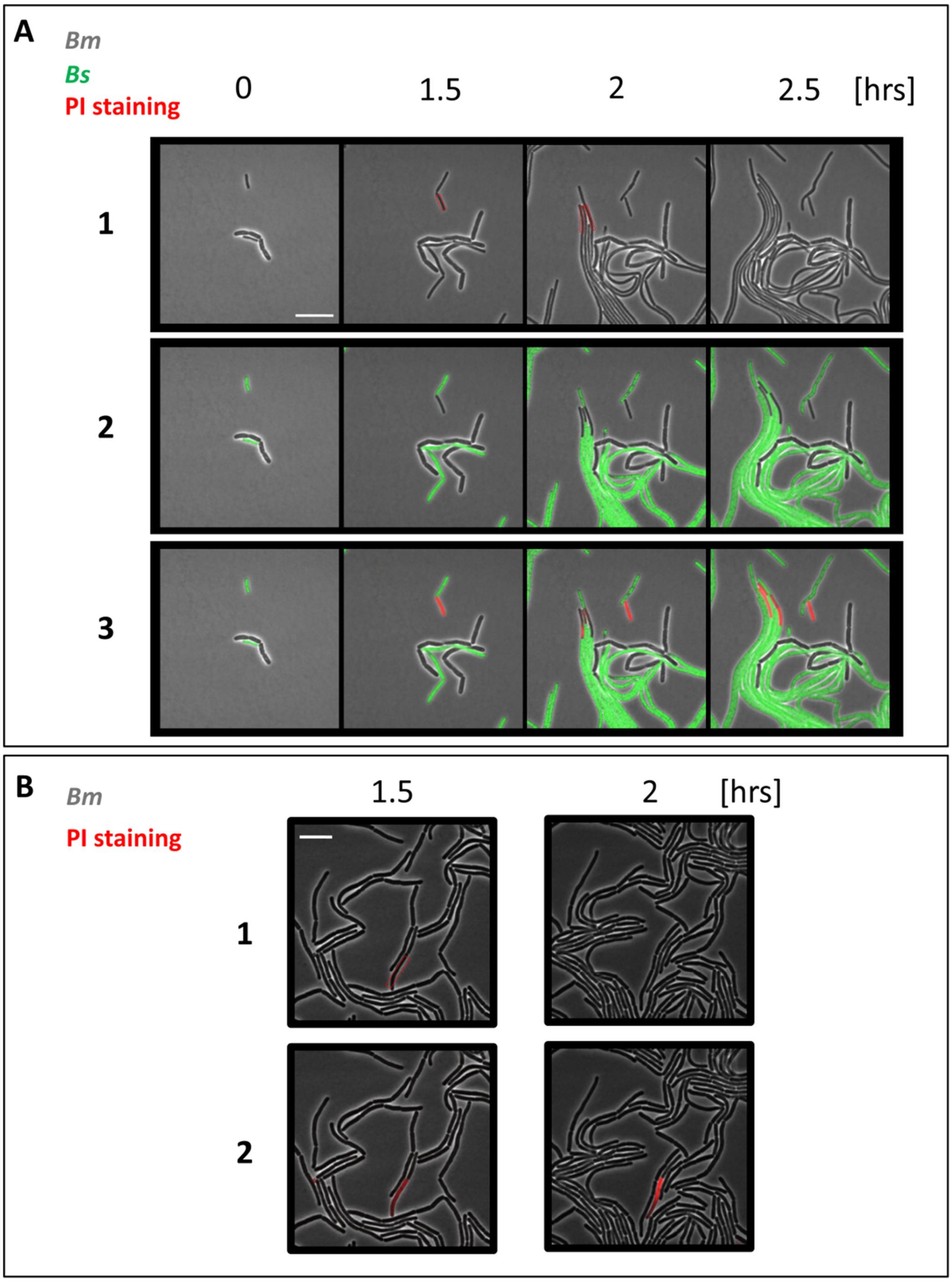


**Supplementary Figure 2: *Bs* toxicity does not perturb *Bm* membrane integrity**

Cells were spotted at low density on solid LB supplemented with propidium iodide (PI) (2 μM) and incubated at 37°C in a temperature controlled chamber and imaged by confocal microscopy. As a positive control, specific cells (marked regions) were irradiated using the 405 nm laser beam at full power (5 mW) and were repeatedly

bleached (50 iterations) in order to observe loss of membrane integrity and PI penetration.

(A) Shown are images of a mixture of *Bs* (AR16: *amyE::*$P_{rrnE}$-*gfp*) and *Bm* (OS2) taken at the indicated time points of (1) phase contrast with red squares indicating the irradiated regions, (2) overlay fluorescence images of GFP (green) and phase contrast (grey), (3) and overlay fluorescence images of GFP (green), PI (red) and phase contrast (grey).

(B) Shown are images of *Bm* (OS2) taken at the indicated time points of (1) phase contrast with red squares indicating the irradiated regions, and (2) overlay fluorescence images of PI (red) and phase contrast (grey).

*Bm* exhibits no fluorescence (black), live *Bs* express GFP (green) and irradiated cells are susceptible to PI staining (red). Scales represent 5 μm.

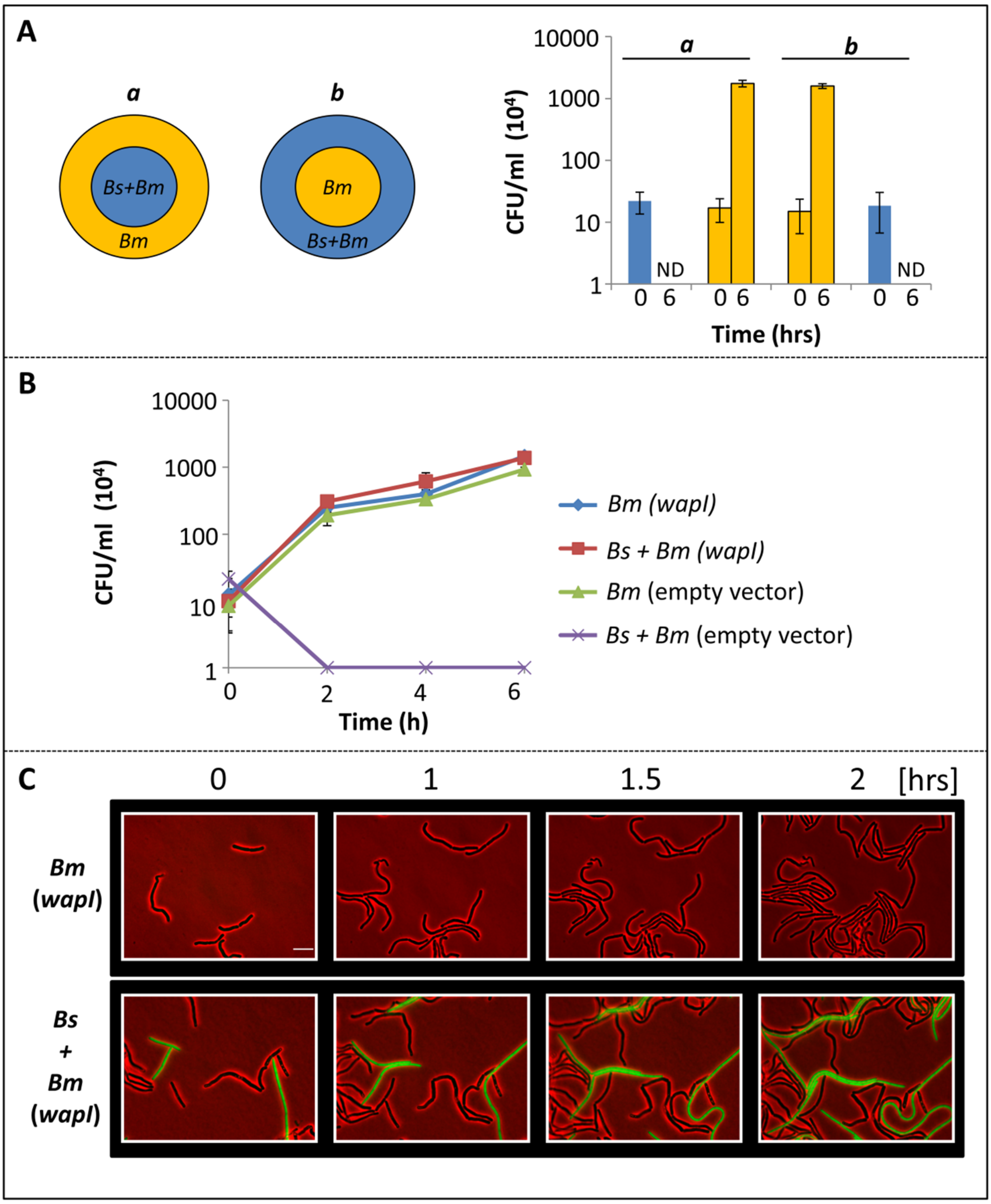


**Supplementary Figure 3: WapI from *Bs* confers immunity to *Bm***

(A) Illustration of a Transwell chamber with mono-culture of *Bm* (OS2) (yellow) and co-culture of *Bm* (OS2) and *Bs* (PY79) (blue). Histogram indicates the growth of *Bm* cells alone or with *Bs* at $t_0$ and after 6 hrs. Each bar represents an average value and SD of three independent experiments. ND-not detected.

(B) *Bm* (pSSBm85-*wapI*) harboring a plasmid containing *wapI* from *Bs*, and *Bm* (pSSBm85) harboring an empty vector were grown separately or in a mixture with *Bs*

(PY79) at 37°C in LB containing xylose to induce *wapI* expression. Cells were plated for CFUs at the indicated time points. Each point represents an average value and SD of three independent experiments.

(C) Representative time lapse microscopy images of *Bm* (pSSBm85-*wapI*) (upper panel) and a mixture of *Bm* (pSSBm85-*wapI*) and *Bs* (AR16: *amyE::*$P_{rrnE}$-*gfp*) (lower panel). Shown are overlay fluorescence from GFP (green) and phase contrast (red) images, captured at the indicated time points. *Bm* cells are shown in black while *Bs* cells are shown in green. Scale bar represents 10 μm.

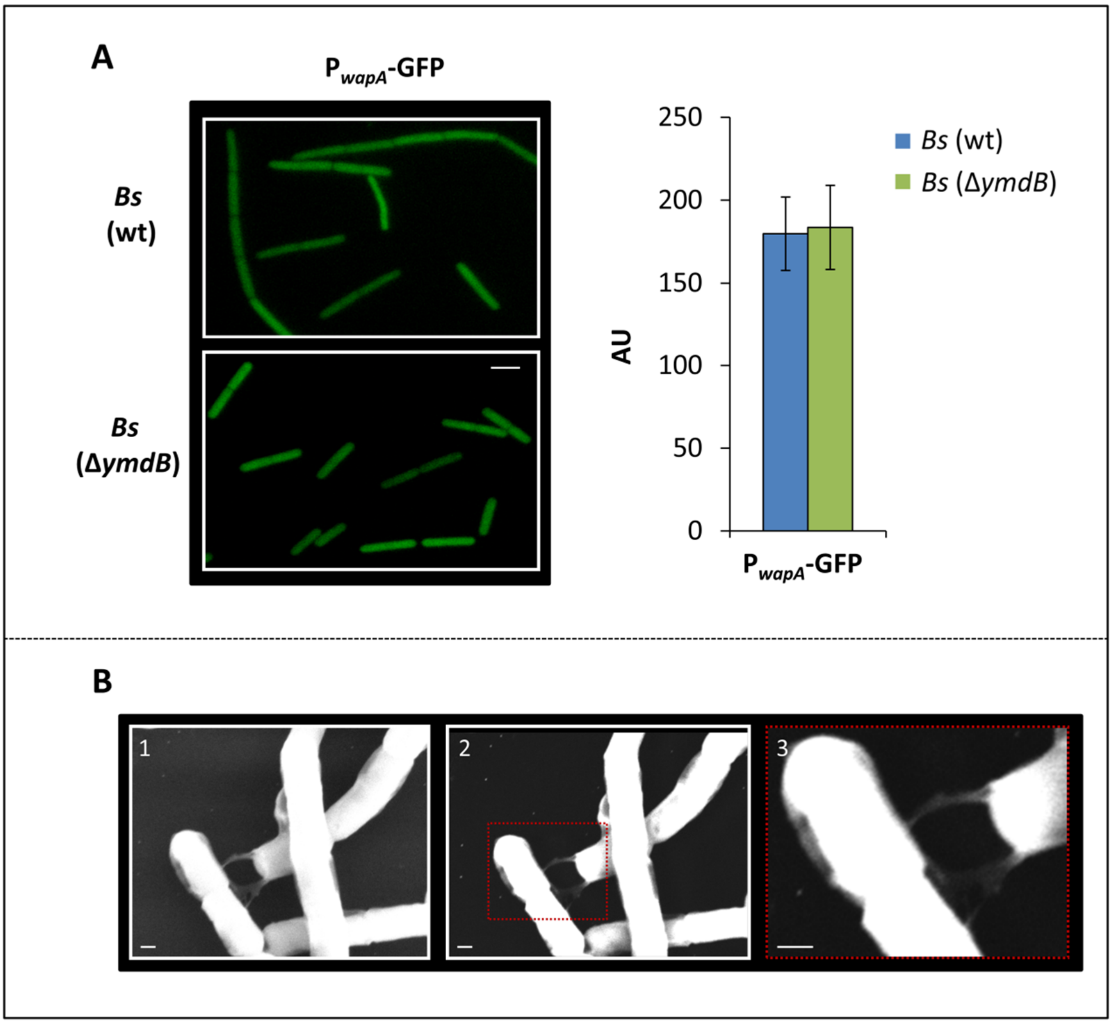


**Supplementary Figure 4: Evidence that YmdB affects WapA delivery**

(A) Representative fluorescence microscopy images of *Bs* (wt) and *Bs* (*ΔymdB*) harboring *amyE::*P$_{wapA}$-*gfp* (left panels), and their average fluorescence in arbitrary units (AU) (right panels). Shown are average values and SD of three independent experiments (n≥ 100 cells). Scale bar represents 5 µm.

(B) *Bs* (GB168: *ΔymdB, Δhag, amyE*::P$_{hyper\text{-}spank}$-*ymdB*) cells, lacking the HA tag, were spotted onto EM grids. Cells were subjected to immuno-gold HR-SEM using primary antibodies against HA and secondary gold-conjugated antibodies. Samples were not coated before observation. Shown are cells connected by intercellular nanotubes. No gold signal (white dots) was obtained from the cells' surface or nanotubes. Some non-specific background gold signal was observed. HR-SEM images were acquired using TLD-SE (1) and vCD (low-kV high-contrast detector) (2). An enlargement of the boxed region in (2) is shown in (3). Scale bar represents 200 nm.

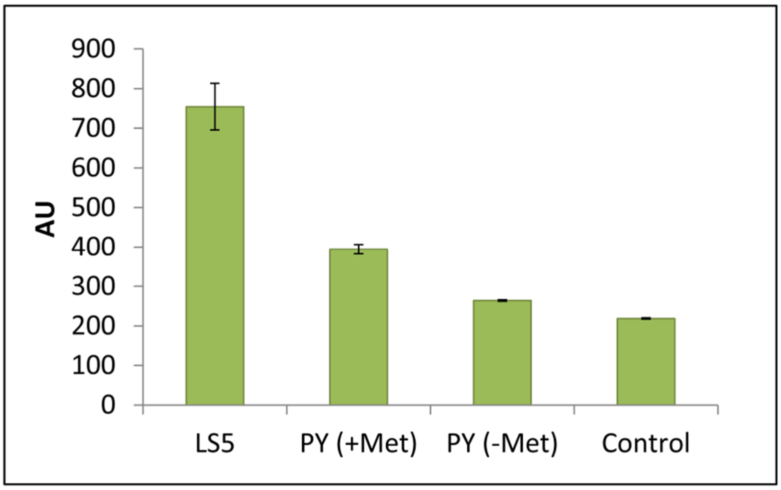


**Supplementary Figure 5: Characterization of AHA uptake by *Bs***

*Bs* (PY79) cells were grown over night in S7 medium in the presence or the absence of Met (50 µg/ml). In addition, *Bs* (LS5: *ΔmetE*) cells were grown in the presence of Met (50 µg/ml). Cells were then washed in PBSx1 and resuspended in S7 supplemented with AHA (1 mM). Cells were incubated for additional 2 hrs, underwent click reaction and visualized by fluorescent microscopy. Shown is quantification of the fluorescent signal emitted from the cells. Control bar represents PY79 cells grown under similar conditions without undergoing the final click reaction. Each bar represents an average value and SD from at least 100 cells.

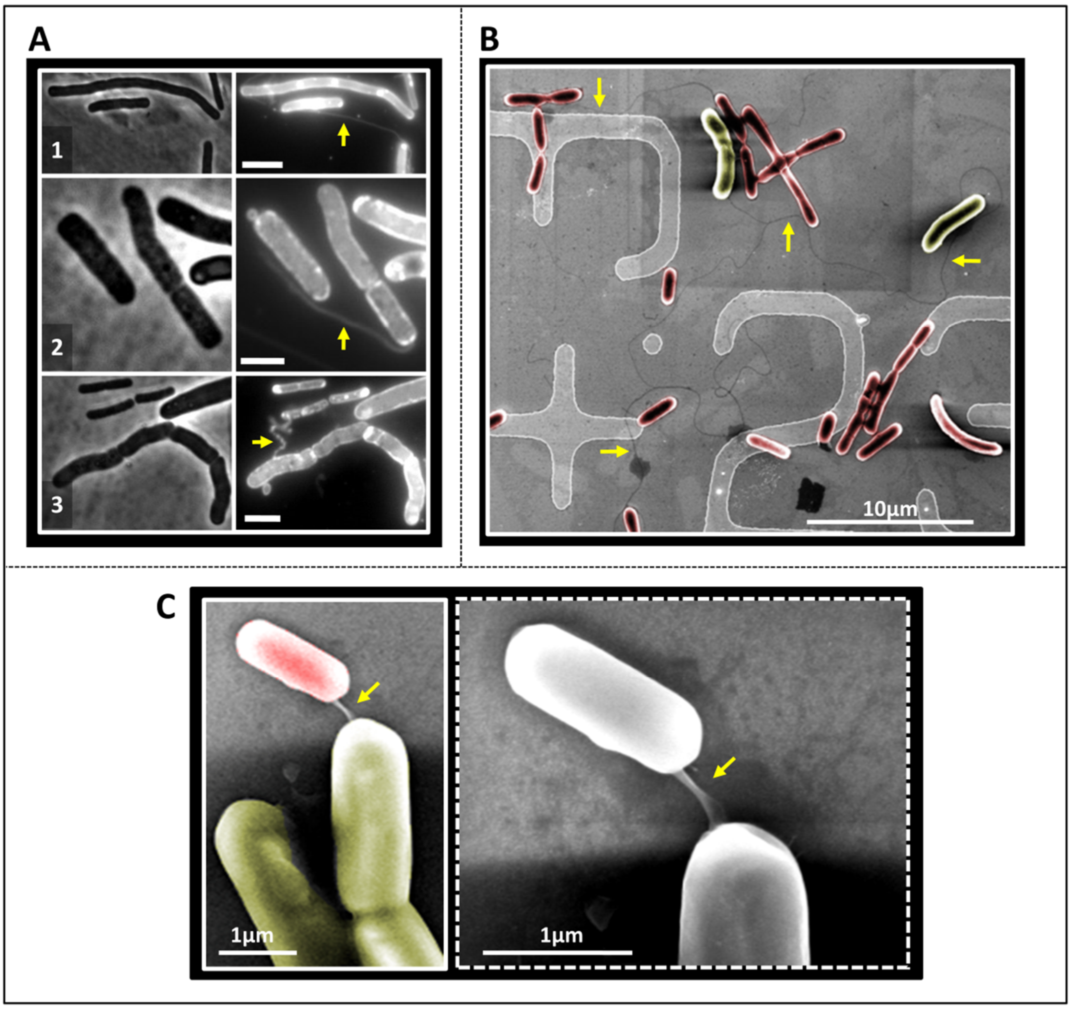


**Supplementary Figure 6: Visualizing intercellular nanotubes linking the two *Bacilli***

(A) *Bs* (PY79) and *Bm* (OS2) cells were spotted at low density onto an ITO-coated cover slip, covered with dialysis membrane and incubated in LB medium supplemented with a fluorescent membrane dye. Shown are phase contrast (left panels) and examples of typical nanotubes observed by fluorescent microscopy (right panels) of (1) *Bs*, (2) *Bm* and (3) interspecies nanotubes. Of note, *Bs* is significantly smaller than *Bm.* Arrows point to nanotube structures. Scale bars represent 2 µm.

(B-C) *Bs* (GB168: *ΔymdB, Δhag, amyE*::P$_{hyper\text{-}spank}$-*ymdB*) and *Bm* (OS2) cells were spotted at low density onto an ITO cover slip, covered with a dialysis membrane, and incubated in LB medium for 1 hr. Cells were then fixed and visualized by HR-SEM without coating. Shown is an HR-SEM image of (B) a nanotube network formed between *Bs* (red) and *Bm* (yellow) (x10,000), and (C) Left: a short nanotube linking two

adjacent cells *Bs* (red) and *Bm* (yellow) (x50,000). Right: is higher resolution (x100,000) of the tube region shown in the left panel. Arrows point to nanotube structures.

**Supplementary Table 1: Investigating the effect of *Bs* mutants on *Bm* growth inhibition**

| Strain name | Mutant gene(s) | Gene function | Reference |
|---|---|---|---|
| GD215 | Δ*hag* | Flagellum subunit | 1 |
| SG157 | Δ*tasA* | Production of an ECM protein | 2,3 |
| GD278 | Δ*tasA*, Δ*hag* | | 4 |
| SB781 | Δ*spo0A* | Master regulator of sporulation, affects biofilm formation. | 5 |
| OS16 | Δ*wapA* | Toxin that was found to cleave tRNases, perturbing translation. | 6 |
| GD413 | Δ*sinR* | Transcriptional regulator, repressor of many biofilm formation genes. | 7 |
| GD420 | Δ*sinI* | Antagonist of SinR | 7 |
| GM3 | Δ*yvfA* | Antagonist of SinR | 8 |
| GM5 | Δ*veg* | Antagonist of SinR | 9 |
| GM9 | Δ*ylbF* | Antagonist of SinR | 10 |
| GB61 | Δ*ymdB* | Phosphodiesterase, affects biofilm formation | 11 |
| GM8 | Δ*yqeK* | Predicted hydrolase involved in multicellularity | 10 |
| GM10 | Δ*yhxB* | Alpha-phosphoglucomutase, involved in multicellularity | 10 |
| GD15 | Δ*yveQ* | Biofilm formation: exopolysaccharide synthesis | 5 |
| SG33 | Δ*yveR* | Biofilm formation: exopolysaccharide synthesis | 5 |
| GD8 | Δ*ywpE* | A putative sortase | 4 |
| GD41 | Δ*yhcS* | A putative sortase | 4 |
| GD43 | Δ*ywpE*, Δ*yhcS* | | 4 |
| GD71 | Δ*ywnE* | Membrane metabolism: cardiolipin synthase | 12 |
| GD73 | Δ*ywjE* | Membrane metabolism: cardiolipin synthase | 12 |
| GD84 | Δ*ywiE* | Membrane metabolism: lipid synthase, involved in cardiolipin synthesis | 12 |
| GD86 | Δ*ypbR* | Membrane metabolism: a putative GTP-binding dynamin like protein | 12 |
| GD87 | Δ*ypfP* | Membrane metabolism: glycosyltransferase | 13 |
| GD88 | Δ*pgsA* | Membrane metabolism: lipid synthase, involved in synthesis of phosphatidylglycerol (Hashimoto et al., 2009) | 14 |
| GD89 | Δ*yerQ* | Membrane metabolism: diacylglycerol kinase, required for lipoteichoic acid production | 15 |
| GD91 | Δ*yfiX* | Membrane metabolism: a putative membrane protein, involved in membrane lipid synthesis | 4 |
| GD70 | Δ*psd* | Membrane metabolism: involved in synthesis of the membrane lipid phosphatidylethanolamine | 16 |
| IB97 | Δ*sigD* | Sigma factor, required for motility, chemotaxis and autolysis. | 17,18 |

## Supplementary Table 2: Bacterial strains and plasmids

| **Species** | **Strain name** | **Genotype** | **Description** |
|---|---|---|---|
| *Bm* | OS2 | Wild type | Soil isolate |
| *Bm* | *Bm*-pSSBm85 | pSSBm85 $tet^R$ | OS2 was transformed with pSSBm85 [19] harboring $tet^R$ gene. |
| *Bm* | *Bm*-pSSBm85-*wapI* | pSSBm85 $tet^R$, $P_{xyl}$-*wapI* | OS2 was transformed with pSSBm85-*wapI* harboring $tet^R$ gene. |
| *Bs* | PY79 | Wild type | [20] |
| *Bs* | AR16 | *amyE::*$P_{rrnE}$*-gfp-spec* | [21] |
| *Bs* | LS5 | *metE::erm* | [22] |
| *Bs* | GB168 | *ymdB::tet, amyE::*$P_{hyper}$*-spank-ymdB- spec, hag::erm* | [23] |
| *Bs* | GB41 | *ΔymdB::spec* | [23] |
| *Bs* | GB61 | *ΔymdB::tet* | [23] |
| *Bs* | BDR2642 | *sacA::*$P_{veg}$*-mCherry* | A gift from D. Rudner (Harvard U) |
| *Bs* | OS16 | *ΔwapA:: spec* | Constructed using Gibson assembly kit (NEB, USA) utilizing primers *wapA::spec*-P1-P4 |
| *Bs* | OS21 | *ΔmetE::erm ΔymdB::spec* | LS5 was transformed with genomic DNA of strain GB41 |
| *Bs* | OS23 | *ΔymdB::tet, sacA::*$P_{veg}$*-mCherry* | GB61 was transformed with genomic DNA of strain BDR2642 |
| *Bs* | OS24 | *sacA::*$P_{veg}$*-mCherry, ΔwapA::spec* | BDR2642 was transformed with genomic DNA of strain OS16 |
| *Bs* | OS25 | *ΔmetE::erm, ΔwapA::spec* | LS5 was transformed with genomic DNA of strain OS16 |
| *Bs* | SH3 | *amyE::*$P_{wapA}$*-gfp-spec* | PY 79 was transformed with pSH2 |
| *Bs* | SH4 | *ΔymdB::tet amyE::*$P_{wapA}$*-gfp-spec* | GB61 was transformed with genomic DNA of strain SH3 |
| *Bs* | SH25 | *wapA*-2×HA*:: cm* | Constructed using Gibson assembly kit (NEB, USA) utilizing primers *wapA*-2×HA-P1-P6 |
| *Bs* | SH29 | *wapA*-2×HA*:: cm ymdB::tet, amyE::*$P_{hyper}$*-spank-ymdB- spec, hag::erm* | GB168 was transformed with genomic DNA of strain SH25 |
| **Plasmids** | | | |
| pSSBm85 | Generously provided by Rebekka Biedendieck (Technische Universität Braunschweig) [19]. | | |
| pSSBm85-*wapI* | *wapI* from *Bs* was amplified from PY79 genomic DNA using primers 3216 wapI-BglII and 3217 wapI-SphI. The amplified DNA was digested with *Bgl*II and *Sph*I and was cloned into pSSBm85 digested with the same enzymes. | | |
| pSH2 | *wapA* promoter region was amplified from PY79 genomic DNA using primers PwapA-F-BamHI and PwapA-R-HindIII. The amplified DNA was digested with *BamH*I and *Hind*III and was cloned into pMM47 [24], containing the *gfp* ORF and the *spec* gene, digested with the same enzymes. | | |

**Supplementary Table 3: List of primers used in this study**

| **Primer name** | **Primer sequence (5' - 3')** |
|---|---|
| 16S rRNA- 7F | AAGAGTTGATCATGGCTCA |
| 16S rRNA- 1511R | GGTTACCTTGTTACGACTT |
| *wapA::spec* - P1 | CAGAGCATACCAATCTGCC |
| *wapA::spec* - P2 | CTGAGCGAGGGAGCAGAATTCCTC<br>TCTCCTTTTGTAATAAAAGTAATA |
| *wapA::spec* - P3 | GTTGACCAGTGCTCCCTGTAAGGTTAAGCGAGGGGGG |
| *wapA::spec* - P4 | CGGGTGTTGGCTGGTATTG |
| 3216 *wapI*-BglII | AAACCCAGATCTATGGCCAAAATAAAAGATGATTGTATAGAAC |
| 3217 *wapI*-SphI | ACCTAGGCATGCAGGAAGTTTAGAAGGATTTTTTGTATTTTC |
| P*wapA*-F-BamHI | AAACCCGGATCC GTAAAAAGGCCTATGCGG |
| P*wapA*-R-HindIII | AAACCCAAGCTT CATTTCCTCTCTCCTTTTGTAATA |
| *wapA*-2×HA - P1 | AAGGCGAAGACAAGCCATTC |
| *wapA*-2×HA - P2 | CATAGGGATAGCCAGCGTAATCTGGAACATCATATGGGTAACG<br>CCTTCTCTTAGATTTATTTCC |
| *wapA*-2×HA - P3 | TTACGCTGGCTATCCCTATGACGTCCCGGACTACGCATAATAA<br>GGTTAAGCGAGGGGGG |
| *wapA*-2×HA - P4 | CTGAGCGAGGGAGCAGAACCCATTTTTATCCAATGCAGA |
| *wapA*-2×HA - P5 | GTTGACCAGTGCTCCCTGATGGGGCATTTATTGATGGA |
| *wapA*-2×HA - P6 | CGTCTTCTACTTCGGGGTCA |
| **qRT-PCR primers** | |
| *wapA* F | CCAGCCGATGTACTAGCAAAATC |
| *wapA* R | GCTTCCTCAGTTTGTTCTTTCTGAAG |
| *rpsE* F | GCGTCGTATTGACCCAAGC |
| *rpsE* R | TACCAGTACCGAATCCTACG |
| 16S rRNA F | CTCGTGTCGTGAGATGTTGG |
| 16S rRNA R | GTTTCGCTGCCCTTTGTTCT |

## Supplementary References


1 Mirel, D. B. & Chamberlin, M. J. The *Bacillus subtilis* Flagellin gene (*Hag*) is transcribed by the Sigma-28 form of RNA-Polymerase. *Journal of bacteriology* **171**, 3095-3101 (1989).

2 Branda, S. S., Chu, F., Kearns, D. B., Losick, R. & Kolter, R. A major protein component of the *Bacillus subtilis* biofilm matrix. *Molecular microbiology* **59**, 1229-1238, doi:10.1111/j.1365-2958.2005.05020.x (2006).

3 Serrano, M. *et al.* A *Bacillus subtilis* secreted protein with a role in endospore coat assembly and function. *Journal of bacteriology* **181**, 3632-3643 (1999).

4 Dubey, G. P. & Ben-Yehuda, S. Intercellular nanotubes mediate bacterial communication. *Cell* **144**, 590-600, doi:10.1016/j.cell.2011.01.015 (2011).

5 Branda, S. S., Gonzalez-Pastor, J. E., Ben-Yehuda, S., Losick, R. & Kolter, R. Fruiting body formation by *Bacillus subtilis*. *Proceedings of the National Academy of Sciences of the United States of America* **98**, 11621-11626, doi:DOI 10.1073/pnas.191384198 (2001).

6 Koskiniemi, S. *et al.* Rhs proteins from diverse bacteria mediate intercellular competition. *Proceedings of the National Academy of Sciences of the United States of America* **110**, 7032-7037, doi:10.1073/pnas.1300627110 (2013).

7 Kearns, D. B., Chu, F., Branda, S. S., Kolter, R. & Losick, R. A master regulator for biofilm formation by *Bacillus subtilis*. *Molecular microbiology* **55**, 739-749, doi:10.1111/j.1365-2958.2004.04440.x (2005).

8 Tortosa, P., Albano, M. & Dubnau, D. Characterization of *ylbF*, a new gene involved in competence development and sporulation in *Bacillus subtilis*. *Molecular microbiology* **35**, 1110-1119, doi:DOI 10.1046/j.1365-2958.2000.01779.x (2000).

9 Lei, Y., Oshima, T., Ogasawara, N. & Ishikawa, S. Functional Analysis of the Protein Veg, Which Stimulates Biofilm Formation in *Bacillus subtilis*. *Journal of bacteriology* **195**, 1697-1705, doi:10.1128/Jb.02201-12 (2013).

10 Branda, S. S. *et al.* Genes involved in formation of structured multicellular communities by *Bacillus subtilis*. *Journal of bacteriology* **186**, 3970-3979, doi:Doi 10.1128/Jb.186.12.3970-3979.2004 (2004).

11 Diethmaier, C. *et al.* A novel factor controlling bistability in *Bacillus subtilis*: the YmdB protein affects flagellin expression and biofilm formation. *Journal of bacteriology* **193**, 5997-6007, doi:10.1128/JB.05360-11 (2011).

12 Lopez, C. S., Alice, A. F., Heras, H., Rivas, E. A. & Sanchez-Rivas, C. Role of anionic phospholipids in the adaptation of *Bacillus subtilis* to high salinity. *Microbiology* **152**, 605-616, doi:10.1099/mic.0.28345-0 (2006).

13 Jorasch, P., Wolter, F. P., Zahringer, U. & Heinz, E. A UDP glucosyltransferase from *Bacillus subtilis* successively transfers up to four glucose residues to 1,2-diacylglycerol: expression of *ypfP* in *Escherichia coli* and structural analysis of its reaction products. *Molecular microbiology* **29**, 419-430, doi: 10.1046/j.1365-2958.1998.00930.x (1998).

14 Hashimoto, M. *et al.* Induction of extracytoplasmic function sigma factors in Bacillus subtilis cells with membranes of reduced phosphatidylglycerol content. *Genes & genetic systems* **84**, 191-198 (2009).

15 Jerga, A., Lu, Y. J., Schujman, G. E., de Mendoza, D. & Rock, C. O. Identification of a soluble diacylglycerol kinase required for lipoteichoic acid production in *Bacillus subtilis*. *J Biol Chem* **282**, 21738-21745, doi:10.1074/jbc.M703536200 (2007).

16 Matsumoto, K. *et al.* Cloning, sequencing, and disruption of the *Bacillus subtilis* psd gene coding for phosphatidylserine decarboxylase. *Journal of bacteriology* **180**, 100-106 (1998).

17 Chen, L. & Helmann, J. D. The *Bacillus subtilis* sigma D-dependent operon encoding the flagellar proteins FliD, FliS, and FliT. *Journal of bacteriology* **176**, 3093-3101 (1994).

18 Cozy, L. M. & Kearns, D. B. Gene position in a long operon governs motility development in *Bacillus subtilis*. *Molecular microbiology* **76**, 273-285, doi:10.1111/j.1365-2958.2010.07112.x (2010).

19 Stammen, S. *et al.* High-Yield Intra- and Extracellular protein production using *Bacillus megaterium*. *Appl Environ Microb* **76**, 4037-4046, doi:10.1128/Aem.00431-10 (2010).

20 Youngman, P., Perkins, J. B. & Losick, R. Construction of a cloning site near one end of Tn917 into which foreign DNA may be inserted without affecting transposition in *bacillus subtilis* or expression of the transposon-borne *Erm* Gene. *Plasmid* **12**, 1-9, doi:Doi 10.1016/0147-619x(84)90061-1 (1984).

21 Rosenberg, A., Sinai, L., Smith, Y. & Ben-Yehuda, S. Dynamic expression of the translational machinery during *Bacillus subtilis* life cycle at a single cell level. *PloS one* **7**, doi:ARTN e4192110.1371/journal.pone.0041921 (2012).

22 Sinai, L., Rosenberg, A., Smith, Y., Segev, E. & Ben-Yehuda, S. The molecular timeline of a reviving bacterial spore. *Molecular cell* **57**, 695-707, doi:10.1016/j.molcel.2014.12.019 (2015).

23 Dubey, G. P. *et al.* Architecture and characteristics of bacterial nanotubes. *Developmental cell* **36**, 453-461, doi:10.1016/j.devcel.2016.01.013 (2016).

24 Meyerovich, M., Mamou, G. & Ben-Yehuda, S. Visualizing high error levels during gene expression in living bacterial cells. *Proceedings of the National Academy of Sciences of the United States of America* **107**, 11543-11548, doi:10.1073/pnas.0912989107 (2010).
